# Phylogenetic and Functional Assessment of Orthologs Inference Projects and Methods

**DOI:** 10.1371/journal.pcbi.1000262

**Published:** 2009-01-16

**Authors:** Adrian M. Altenhoff, Christophe Dessimoz

**Affiliations:** Institute of Computational Science, ETH Zurich, and Swiss Institute of Bioinformatics, Zürich, Switzerland; University of California Davis, United States of America

## Abstract

Accurate genome-wide identification of orthologs is a central problem in comparative genomics, a fact reflected by the numerous orthology identification projects developed in recent years. However, only a few reports have compared their accuracy, and indeed, several recent efforts have not yet been systematically evaluated. Furthermore, orthology is typically only assessed in terms of function conservation, despite the phylogeny-based original definition of Fitch. We collected and mapped the results of nine leading orthology projects and methods (COG, KOG, Inparanoid, OrthoMCL, Ensembl Compara, Homologene, RoundUp, EggNOG, and OMA) and two standard methods (bidirectional best-hit and reciprocal smallest distance). We systematically compared their predictions with respect to both phylogeny and function, using six different tests. This required the mapping of millions of sequences, the handling of hundreds of millions of predicted pairs of orthologs, and the computation of tens of thousands of trees. In phylogenetic analysis or in functional analysis where high specificity is required, we find that OMA and Homologene perform best. At lower functional specificity but higher coverage level, OrthoMCL outperforms Ensembl Compara, and to a lesser extent Inparanoid. Lastly, the large coverage of the recent EggNOG can be of interest to build broad functional grouping, but the method is not specific enough for phylogenetic or detailed function analyses. In terms of general methodology, we observe that the more sophisticated tree reconstruction/reconciliation approach of Ensembl Compara was at times outperformed by pairwise comparison approaches, even in phylogenetic tests. Furthermore, we show that standard bidirectional best-hit often outperforms projects with more complex algorithms. First, the present study provides guidance for the broad community of orthology data users as to which database best suits their needs. Second, it introduces new methodology to verify orthology. And third, it sets performance standards for current and future approaches.

## Author Summary

The identification of orthologs, pairs of homologous genes in different species that started diverging through speciation events, is a central problem in genomics with applications in many research areas, including comparative genomics, phylogenetics, protein function annotation, and genome rearrangement. An increasing number of projects aim at inferring orthologs from complete genomes, but little is known about their relative accuracy or coverage. Because the exact evolutionary history of entire genomes remains largely unknown, predictions can only be validated indirectly, that is, in the context of the different applications of orthology. The few comparison studies published so far have asssessed orthology exclusively from the expectation that orthologs have conserved protein function. In the present work, we introduce methodology to verify orthology in terms of phylogeny and perform a comprehensive comparison of nine leading ortholog inference projects and two methods using both phylogenetic and functional tests. The results show large variations among the different projects in terms of performances, which indicates that the choice of orthology database can have a strong impact on any downstream analysis.

## Introduction

The identification of orthologs is an important problem in the field of comparative genomics. Many studies, such as gene function prediction, phylogenetic analyses, and genomics context analyses, depend on accurate predictions of orthology. A large variety of methods for predicting orthologs and the resulting databases have appeared in recent years [1]–[8]. But although the accuracy of the predictions highly impacts any downstream analyses, there are only few comparative studies of the quality of the different prediction algorithms [9],[10]. This paucity can be attributed to at least three major challenges. The first challenge resides in the multiple and sometimes intrinsically conflicting definitions of orthology [11]–[13]. The original definition of Fitch [14] is based on the evolutionary history of genes: two genes are orthologs if they diverged through a speciation event. On the other hand, given that orthologs often have similar function, many people uses the term orthologs to refer to genes with conserved function. Yet another definition is used in some studies of genome rearrangement, in which the ortholog refers, in the event of a duplication, to the “original” sequence [15], which remains in its genomic context.

The second challenge resides in the difficulty of validating the predictions. Take the case of phylogenetic orthology. Gene tree inference can be a notoriously difficult task, but it is usually precisely in difficult cases that the performances of methods can be differentiated. Indeed, in simple cases, most methods perform equally well. Validation of the definition based on function is not easier: orthology is in this context arguably *impossible* to verify because there is no universally applicable, unequivocal definition of conserved function, that is, the required similarity in terms of regulation, chemical activity, interaction partners, etc. for two genes to qualify as orthologs often varies across studies. For instance, in some wet lab experiments [16],[17], two genes are only considered orthologs if they have the ability to complement each other's function.

The third challenge is of practical nature: to compare the different orthology inference projects, their methods must either be replicated on a common set of data, or the results produced by the different databases must be mapped to each other for comparison. Replication is not always possible, because some projects depend on human curation, or are not documented in detail. Mapping data is complicated by the lack of homogeneity in the sources of genomic data used by the different projects. The resulting intersection sets are often relatively small and may not be representative.

In the present article, we provide an in-depth comparison of the prediction from 11 major projects, including OMA [4], our own orthology inference effort. We try to address the aforementioned challenges by testing phylogenetic and functional definitions of orthologs, using a variety of tests. We took the approach of comparing the inferred orthologs available from the different projects, which required mapping the data between projects. The rest of this introduction provides a description of the projects retained here, a review on the representation of orthology in those projects so to provide a common basis for comparison, and finally, some words on our sequence mapping strategy.

### Projects under Scrutiny

**Figure 1:**
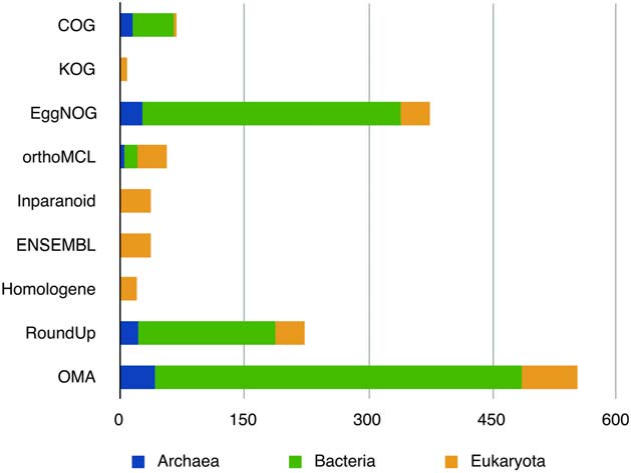
Number of complete genomes analyzed by the different projects.

In this study, we consider publicly available databases of orthologs that distinguish themselves by popularity, size, quality, or methodology. One of the oldest large-scale orthology database is COG [1],[18] and its eukaryotic equivalent KOG [18], which despite no recent update are still considered by many authors as the standard orthologs databases. Their reliance on manual curation make them not scalable to all complete genomes. Unsupervised orthology assignment requires more sophisticated algorithms, such as those of Inparanoid [2],[19], OrthoMCL [3] or EggNOG [8]. We also investigated the results of RoundUp [5], interesting for its relatively large size and its use of pairwise evolutionary distances between genes to detect orthology. OMA [4],[20], our own orthology assignment project, is also based on evolutionary distances but takes into account the variance of the distance estimates and try to exclude pseudo-orthologs arising from differential gene losses using third-party species. A very different approach is taken in the orthology prediction of Ensembl Compara[7], which is based on inference and reconciliation of gene and species trees. Homologene [6] uses a pairwise gene comparison approach combined with a guide tree and gene neighborhood conservation to group orthologs, but the details of their methodology are unpublished. Finally, we also compare the results to the standard approaches of bidirectional best-hits (BBH) [21], common in ad-hoc analyses, and reciprocal smallest distance (RSD) [22]. The size of the different projects is depicted in Figure 1.

### Grouping of Orthologs

Orthology is a relation over pairs of genes. However, few projects (namely Ensembl Compara, OMA and RoundUp) explicitly provide output of all pairs of predicted orthologs. This representation, although precise, has practical drawbacks: on one hand, it scales poorly (quadratically with the number of genes analyzed), and on the other hand, it does not present the predictions in a particularly insightful way. To solve these issues, many projects cluster pairs of orthologs into groups. This grouping process is not trivial, because orthology, at least when the phylogeny-based definition applies, is a non-transitive relation.

The most common approach (taken by all other projects) is to form groups of orthologs and “in-paralogs”. The relations in- and out-paralogs were defined by Remm *et al.* [2], and are used to distinguish between paralogs from recent and old duplication events respectively. Formally, these two relations are not defined over a pair, but over a triplet: two genes and a speciation event of reference. Two genes are in-paralogs with respect to a particular speciation event if they are paralogs *and* their duplication event occurred after that speciation event of reference. They are out-paralogs if they are paralogs *and* their duplication event occurred before the speciation event of reference. See Figure S1a in *Supporting Information* for an example. Unfortunately, the fact that in- and out-paralogy are ill-defined in the absence of a clear speciation event of reference is underappreciated in the literature. We now come back to the description of groups of orthologs and in-paralogs: such groups are constructed such that every pair of genes in the group is either orthologous or in-paralogous with respect to the last speciation event in their clade, that is, such in-paralogs are genes inside the same species resulting from a duplication event that occurred *after* all speciation. Consequently, in such groups, the implication is that gene pairs are orthologs if they belong to the different species, else they are paralogs. Note that this grouping approach shows its limits when one or several duplication events have occurred after the first, but before the last speciation events. In such cases, not uncommon in Eukaryotes, the non-transitive nature of orthology makes it impossible to partition all genes in such groups without losing orthologous relations (see Figure S1b for an example). In OMA for instance, groups of orthologs include less than half of all predicted pairwise orthologous relations (Table S1). This problem does not affect Inparanoid, because it provides predictions for each pair of species separately, and so in every case, there is only one speciation event.

### Mapping Strategy

To perform a fair comparison of the different predictions, a common set of sequences must be established. Unfortunately, the different projects vary considerably in their sizes, the type of genome analyzed and the origin of the protein sequences used. In fact, some projects have no overlap at all, and therefore comparison on a common set of sequences for all projects is not possible. Instead, we performed pairwise project comparisons with OMA (which includes the largest amount of sequences), and then we repeated the tests on an intersection set with only the most competitive projects.

First, sequences from the different projects were mapped to OMA's only if they were identical, between consistent genomes. This strict requirement avoids reliance on IDs, which may refer to different sequences depending on the genome version, and also the problem of different splicing variants. Tables S1 and S2 in *Supporting Information* present some statistics on the mapping procedure of the sequences and the predictions.

In pairwise tests, we compared the pairs of mappable proteins identified as orthologs by the different methods with those identified by OMA. In joint tests, we computed the intersection of the mappable sequences of each project under consideration, and compared pairs in this intersection set identified as orthologs by the different methods. Datasets S1, S2, S3, S4, S5, S6, S7, S8, S9, S10, S11, S12 in *Supporting Information* list the intersection sets we used in all analyses below.

## Results/Discussion

In this section, we present all results, first in pairwise comparisons between each project and OMA, then in joint comparisons of the most competitive projects. We group the tests according to the definition of orthology that they should verify: the first two tests verify orthology based on phylogeny, while the four following tests verify orthology based on on function. At the end of the section, we justify the absence of tests that were not included here, and compare our results with the previous study of Hulsen *et al.* [9].

### Phylogeny-Based Definition

According to the phylogenetic definition, two homologous genes are orthologs if they diverged through a speciation event. Therefore, the phylogenetic tree of a set of orthologs (a set of genes in which any pair is orthologous) has by definition the same topology as the corresponding species tree.

#### Gene tree reconstruction

We reconstructed gene trees from species with an accepted phylogeny and predicted orthologs from the different projects using two independent methods and software packages (distance-trees from Smith-Waterman pairwise alignments and ML trees from multiple sequence alignments), and compared the congruence of the resulting trees with the species trees using the fraction of correct splits, which is defined as one minus the Robinson-Foulds (RF) split distance measure [23]. The RF distance is defined as the normalized count of the bipartitions induced by one tree, but not by the other. The experiment was performed on sets of bacteria, of eukaryotes and of fungi. Note that this test can only verify the correctness of the reported orthologs (the specificity) for each project, but not the false-negative rate (the sensitivity).

**Figure 2:**
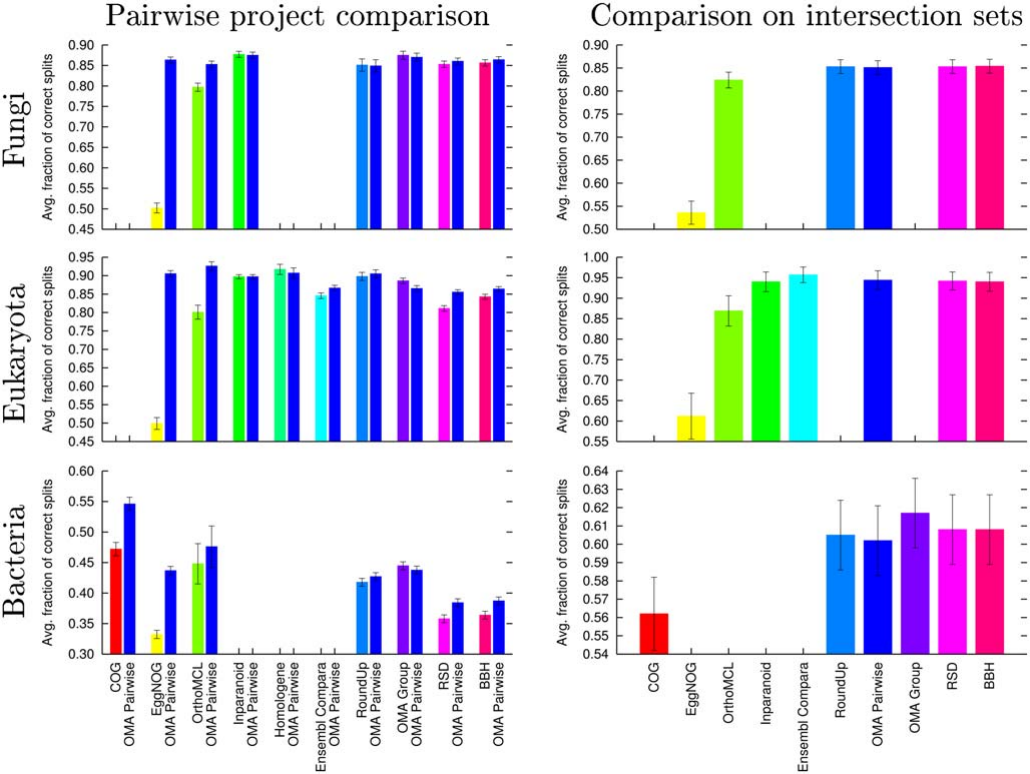
Results of phylogenetic tree test. The mean fraction of correct split of ML trees for gene trees from three different kingdoms are shown. The higher the values, the better the gene trees agree with the species tree. On the left, the pairwise results between every project and OMA are shown, whereas on the right, the result for the comparison on the common set of proteins of a larger number of projects is shown. Note that the pairwise project comparisons are made based on varying protein sets, and thus can not be compared to each other. Error bars indicate the 95% confidence intervals of the estimated means. Projects with too little appropriate data could not be evaluated, which explains absent bars.

Though some level of incongruence is expected from errors in the input data or in the tree reconstruction, these perturbations affect, on average, all methods equally. Results for ML trees are presented in Figure 2 while distance trees are presented in Figure S2 in *Supporting Information*. As a first observation, it is comforting to see that the choice of tree reconstruction method does not affect the ranking or the significance of the results. It appears that COG, EggNOG and OrthoMCL suffer from comparatively high false-positive rates, which is reflected in the significantly reduced amount of correctly reconstructed gene trees. The high-level of non-orthology in the COGs database is consistent with previous reports [24],[25]. The differences among the better performing projects are small. The predictions of Ensembl Compara, being made on the basis of tree reconciliation, could have been expected to perform better than pairwise gene comparison methods, but their predictions are in fact slightly worse than OMA in this test. The generic BBH and RSD methods are also dominated by OMA in the pairwise comparison. Note that the intersection set is not large enough to allow the ranking of the best performing projects (OMA, RoundUp, Homologene, Inparanoid). Finally, KOG covers too few genomes for inclusion in this test.

#### Benchmarks from literature

**Figure 3:**
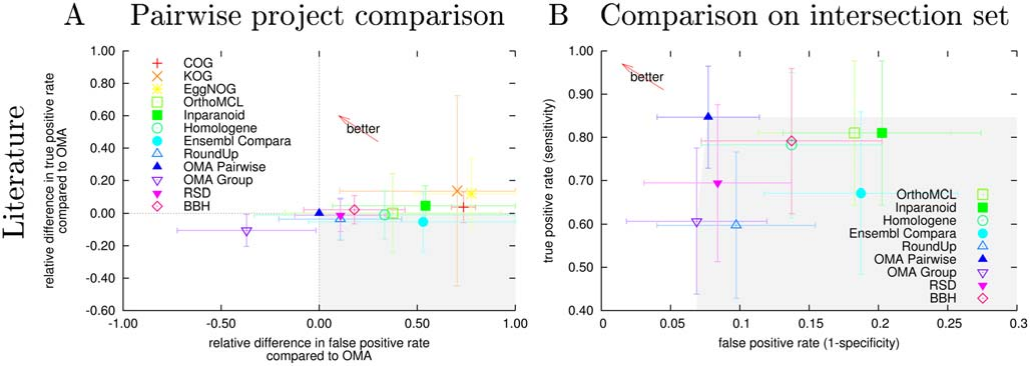
Results of benchmarks from literature. Performance on manually curated gene trees from 4 published studies. [9],[24],[26],[27]. (A) The pairwise outcome of every project against OMA are shown, indicated with the relative difference of the true positive rate between OMA and its counter project versus their relative difference of the false-positive rate. (B) Performance for the protein intersection dataset. Shown are the true positive rate (sensitivity) versus the false-positive rate (1 - specificity). In both plots, the error bars indicate the 95% confidence interval and the “better arrow” points into the direction of higher specificity and sensitivity. Projects lying in the gray area are dominated, in (A) by “OMA Pairwise” and in (B) by at least one other project.

The accuracy of the different projects in terms of the phylogeny-based definition of orthology was also assessed from manually curated gene trees or reference orthology sets from four studies [9],[24],[26],[27]. In addition, this method allows us also to estimate the true positive rate (sensitivity) of the different projects, that is, the fraction of reported orthologs over all bona fide orthologs. Figure 3 summarizes the performance of the projects on those difficult phylogenies. In the pairwise project comparison (Figure 3A), the relative difference between the true positive rate of OMA and the comparative project versus their relative difference of the false-positive rate is shown. Strictly speaking, only pairwise comparisons with OMA should be made, since the underlying protein sets are not the same across different projects and thus, the difficulties of prediction may differ. On the other hand, Figure 3B compares a selection of the projects on a common set of sequences. The results for projects analyzed in both contexts have good agreement, which suggest that pairwise comparisons (which are based on more data) also provide a global picture across projects. The confidence interval around the points are relatively large, due to the limited data used in this test.

First, COG/KOG/EggNOG show higher sensitivity (true positive rate), but at the cost of very low specificity (high false-positive rate). This is a clear sign of excessive clustering. It also appears that the relatively higher false-positive rate of OrthoMCL is not compensated by a significantly higher true-positive rate. Ensembl and RoundUp report fewer orthologs, but the accuracy of their predictions is not significantly higher than OMA or even BBH. Inparanoid, with its relatively low specificity, is doing worse than in the previous test. But overall, the agreement with the previous test in terms of false-positive rate is good, even though the testing methodology is here very different.

### Function-Based Definition

One of the main application of orthology is the propagation of functional annotation, because orthologs often have a similar function. In fact, this application is so prominent that many authors use the term “orthologs” to refer to genes with conserved function in different species. As mentioned in the introduction, this definition is ambiguous. Therefore, we could only test specific aspects of what can be implied by “conserved function”.

The four tests presented here evaluate the similarity of predicted orthologs in terms of gene ontology annotations, enzyme classification numbers, expression level, and gene neighborhood conservation. In the following, we present and discuss their results.

#### Gene ontology

In the first test, we assessed the agreement in gene ontology (GO) annotations [28] between predicted orthologs, only considering annotations with experimental support (Evidence codes IDA, IEP, IGI, IMP and IPI). Indeed, annotation obtained automatically are for the most part done using the methods that we are testing here: inclusion of this information would cause a serious circular dependency. We measure the level of conservation in terms of GO annotation using the similarity measure developed by Lin [29] which computes for a pair of terms a score between 0 (unrelated) and 1 (identical terms) using the hierarchical structure of the GO terms and their frequencies.

**Figure 4:**
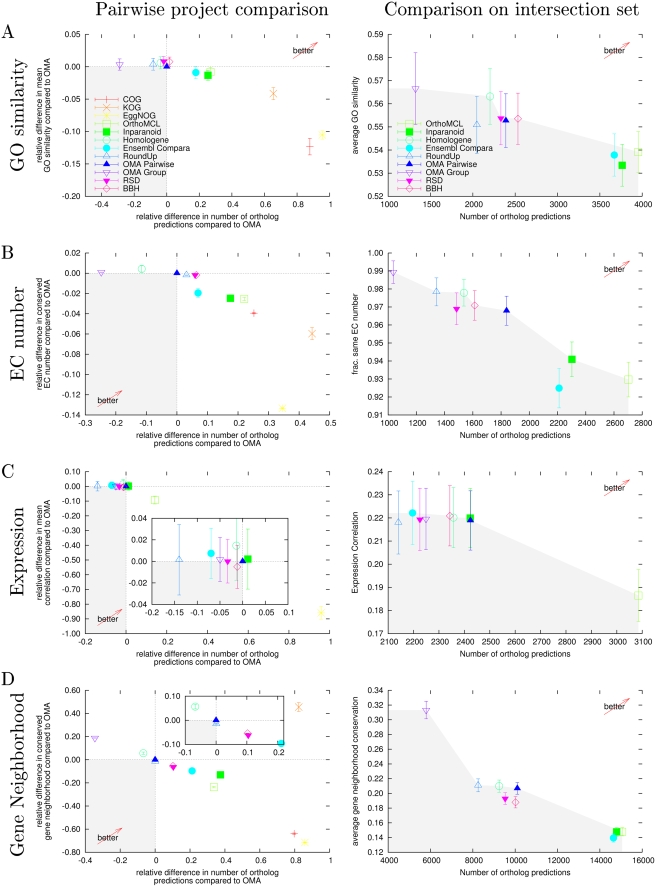
Results of functional based tests. Results of functional conservation tests for GO similarity, EC number expression correlation and gene neighborhood conservation. In the pairwise project comparisons (left) the relative difference of functional similarity between OMA and its counter project versus the relative difference of the number of predicted orthologs are shown. In the comparison on the intersection set (right), the mean functional similarity versus the number of predicted orthologs on the common set of sequences are shown. The vertical error bars in all the results state the 95% confidence interval of the means. The “better arrow” indicates the direction towards higher specificity and sensitivity. Projects lying in the gray area are dominated by “OMA Pairwise” in the pairwise comparison (left) and by at least one other project in the intersection comparison (right).

Figure 4A shows the average similarity of GO annotations in pairs of orthologs from the different projects. The mean similarity of all projects falls in a relatively small range, and is quite low. COG/KOG/EggNOG do comparatively many predictions, but the average similarity score is significantly lower. Hence, the results of COG/KOG/EggNOG are particularly suited for coarse-grained functional classification. On the other hand, if a high functional similarity is desired, the relatively simple BBH approach dominates more sophisticated algorithms such as RoundUp and Homologene (which does fewer predictions at same degree of similarity) or OMA (which does only few more predictions, but significantly lower degree of similarity). This result suggests that sequence similarity is a stronger predictor of functional relatedness than the evolutionary history of the genes. At mid specificity level, OrthoMCL outperforms Ensembl Compara and Inparanoid, yielding many more predictions at roughly the same similarity level.

#### Enzyme classification

A second measure for the quality of the orthologous assignments with respect to function can be obtained from the enzyme classification numbers (EC), which strictly depend on the chemical reaction they catalyze. Thus, we could expect in general that orthologous enzymes have identical EC number. Obviously, this test can only be applied to the small and rather specific fraction of genes that are enzymes. The results must be interpreted accordingly. As reference, we use the EC database curated by the Swiss-Prot group [30]. Their annotation is a semi-supervised procedure that mainly relies on sequence similarity (Kristian B Axelsen, personal communication). As such, this test is less reliable than the GO one, which is based on fully orthogonal data, but we believe that it has enough informative value to warrant inclusion here.

Figure 4B shows the difference between the projects. The results are very similar to the GO annotations test, but BBH is not as good, and Inparanoid has now moved to the Pareto frontier, i.e. it is not dominated by OrthoMCL here.

#### Correlation in expression profiles

In this third test, conserved function is assessed using protein expression profiles from large-throughput experiments. In such data, proteins with similar function are expected to have similar expression profiles. We measured this similarity by computing the average correlation between the expression profiles of putative orthologs between the human and mouse genomes as presented by Liao and Zhang [31]. Some projects, such as COG and KOG did not have sufficient mappable proteins in those genomes to be considered here. Although certainly relevant for many researchers, Human–Mouse orthologs hardly constitute a representative sample of all orthologs, and thus here too their assessment should be extrapolated to all predictions with prudence.

The results are shown in Figure 4C. In general, the correlations found are relatively low and within a narrow band. This range is however consistent with the results of Liao and Zhang. Most projects perform very similarly, with average correlation mostly within 2 standard deviations and number of predicted orthologs differing by less than 10%. Predictions by OrthoMCL have significantly lower average expression correlation, but in absolute terms, the difference is modest, and they have a significantly higher number of predictions. Finally, with 40 times more predictions but almost no correlation in terms of expression, EggNOG does not appear to provide useful information to propagate expression levels.

#### Gene neighborhood conservation

To assess the quality of the ortholog assignments on the basis of genome structure, conservation of the gene arrangement on the chromosomes has been used to validate functional orthology in previous studies [9],[25],[32]. Conservation of the genomic context is indeed a strong indicator of function conservation. Note that gene neighborhood conservation is not a reliable indicator of phylogenetic orthology: not only speciation, but also duplication of DNA segments stretching over more than a single gene, such as operons, preserve the immediate neighborhood.

In this test, we measure the fraction of orthologs that have at least one pair of flanking orthologs (see *Methods*). The results are presented in Figure 4D. The pairwise project comparison shows results consistent with previous tests, with the exception of KOG, which appears to perform extremely well in the pairwise test with OMA. However, the results are based on relatively few and distant genomes that have low absolute conservation values (see raw data in Text S2 of *Supporting Information*). In such a context, the much larger number of ortholog predictions of KOG significantly increases the probability of having adjacent pairs of orthologs due to chance only.

In terms of methodology, Homologene is the only project that uses gene neighborhood conservation as part of their methodology. The details of how precisely such information is exploited in their inference process remain unpublished, but the present test does not show significant improvement over other approaches in terms of neighborhood conservation.

### About Absent Tests

We now justify the absence of three other tests that have been previously reported in the literature. We did not verify orthology based on common keywords in the annotation because those are often assigned on the basis of sequence similarity or using the methods that are tested here: this would introduce circularity in the testing strategy. Nor do we test orthology based on conservation in protein-protein interaction (PPI). Though there are studies such as Bandyopadhyay *et al.* [33] reporting modest but measurably higher PPI between some orthologs, it remains unclear to us how current PPI data can be turn into a test of orthology for the following two reasons: first, PPI data show large variations in reliability and completeness across experiments and species, but more importantly, the general problem of matching (or “aligning”) networks is computationally hard [34]. To reduce complexity, most approaches, including Bandyopadhyay *et al.* [33], strongly constrain the network alignment using heuristics based on sequence similarity. In the present context, this too would introduce circularity in the validation. Finally, we do not use the latent class analysis approach of Chen *et al.* [10]. This approach computes maximum likelihood estimates of false-positive and false-negative rates for all the projects directly from the various ortholog predictions (the data) and a parameterized multivariate distribution of the errors (the model). This looks very attractive, because the assessment does not require any of the external information used in the tests described here. Our critique with this approach is that their results are conditional on their error model, which is not verified (at least not in the context of evaluating orthology inference projects). In a sense, the issue of validation is shifted to their error model, but remains open.

### Comparison with Results of Hulsen *et al.*

The main other systematic evaluation of orthology prediction projects is from Hulsen *et al.* [9]. Smaller in scope, their study tested functional orthologs predictions in Human–Mouse and Human–C. elegans, using a manually curated reference set of orthologs, expression correlation and conservation of gene neighborhood. They compared BBH, Inparanoid, OrthoMCL, KOG, as well as two other methods not under analysis here (“PhyloGenetic Tree” and “Z 1 Hundred”).

On the tests and data common to both studies, the results are largely consistent (data not shown). However, we observed that considering only two pairs of species can introduce significant biases in the assessment: as it turns out, the overwhelming majority (89.1%) of all orthologous pairs predicted by Inparanoid on Human–Worm data arise from one large cluster of olfactory-type receptor proteins (cluster number 4604). This very atypical distribution explains why the results are so different from those for the HUMAN-MOUSE genome pair (see Figures 3 and 4 from [9]).

They concluded that in terms of functional orthology, Inparanoid performed best overall, while also noting that the appropriate method depends on the user's requirements in terms of sensitivity and specificity. As our results show, this trade-off remains true today, but Inparanoid is no longer the overall best performer: besides being one of the projects with fewest genomes under analysis, there are other projects with either higher specificity, or with higher sensitivity; this reduces the scope of applications in which it constitutes an appropriate choice.

### Conclusions

Accurate ortholog prediction is crucial for many applications ranging from protein annotation to phylogenetic analysis. There is a number of publicly available orthology databases but little is known about their performances. In this study we compared 11 different projects and methods by submitting them to a variety of tests with respect to both phylogenetic and functional definitions of orthology.

The results obtained in the tests for both definitions are consistent, and allow us comparison of the different projects on an objective basis.

In phylogenetic tests, OMA and Homologene showed the best performances. The same two projects do also best in functional tests if a high level of specificity is required. At a somewhat lower degree of specificity, but at a higher coverage, function-based tests suggest that OrthoMCL outperforms Ensembl Compara, and to a less extent Inparanoid. Finally, for applications that only require coarse-grained functional categories, EggNOG provides the largest coverage.

In terms of methodology, the one project based on gene and species tree reconciliation, Ensembl, had overall decent performances, but was overperformed by some of the best pairwise approaches. This suggests that tree reconciliation, although more powerful a method in theory, is not necessarily the best method in practice. Another surprise is the good overall performance of the simple BBH approach. Although the method is restricted to 1:1 orthologs, the derived relations show good comparative accuracy in terms of Fitch's definition. Orthologs predicted by BBH also show close functional relatedness. This result probably explains why many people use ad-hoc BBH implementations for their analyses rather than a more sophisticated orthology method.

Beyond the accuracy aspects discussed in the present work, other factors will also affect the choice of orthologs database, such as the number of genomes analyzed, the state of maintenance, the availability of the predictions, or the usability of the web-interface.

There is still improvement potential in orthology inference, and we expect much development in the coming years. We hope that the present work helps setting performances standards. But it is also the responsibility of upcoming orthology assignment projects or releases to clearly state the definition of orthology they pursue, to explain their grouping strategy, and in the very least to demonstrate the improvement of their methods over basic methods such as BBH or RSD.

## Methods

### Input Data

All the projects included in this study are publicly available. A short description of the chosen configurations and references are given in the following. We used the default parameters unless mentioned otherwise.

**RoundUp:** RoundUp can be downloaded from https://rodeo.med.harvard.edu/tools/roundup/. It is available with different parameter settings to tune for the desired sensitivity. In this comparison we included the strictest parameter set (also default settings), i.e. Blast E-value cutoff 10^−20^ and divergence cutoff 0.2.

**Inparanoid:** Inparanoid is available from http://inparanoid.sbc.su.se. We used the release 6.0 from Aug 2007 including 35 species.

**Ensembl Compara:** The orthology predictions from Ensembl were obtained from the Compara database version 47, which is available from http://oct2007.archive.ensembl.org/.

**COG,KOG:** Cluster of Orthologous Groups and its eukaryotic equivalent are available from http://www.ncbi.nlm.nih.gov/COG/. We used the versions from Mar 2003 and Jul 2003 respectively.

**OrthoMCL:** We obtained the version from Sep 2006 of OrthoMCL from http://orthomcl.cbil.upenn.edu/.

**Homologene:** Homologene is available from the NCBI webpage www.ncbi.nlm.nih.gov/HomoloGene/. For this comparison, we used built 58 from Nov 2007.

**EggNOG:** EggNOG is available from http://eggnog.embl.de/. We used the data from Oct 2007 including 373 species.

**OMA:** OMA is available in various formats on http://www.omabrowser.org. We used the the data from Nov 2007 including 550 species. OMA infer orthology at the level of pairs of sequences (“OMA Pairwise”), from which it also computes groups of orthologs (“OMA Group”). Both type of predictions are included in the comparisons.

**BBH:** The typical Bidirectional Best Hit implementation uses BLAST for aligning the protein sequences. We used the more accurate algorithm from Smith and Waterman [35] for the alignment with the same scoring threshold as used by the OMA algorithm for the all-against-all step.

**RSD:** Reciprocal Smallest Distance orthology relations are computed using ML distance estimates from pairwise alignments having significant alignment scores (Dayhoff score >217, the cut-off used by OMA as well)

### Phylogenetic Reconstruction Test

A consequence of Fitch's definition is that trees of orthologs are congruent to the species tree (i.e. the topology, or branching order, is the same). The phylogenetic reconstruction test uses this property to test the predicted orthologs. It uses three reference species trees (see Text S1 in *Supporting Information*) whose branching order is well-accepted, and whose topology follows a “comb” structure, that is, completely unbalanced. Each leaf consists of one or several species. The phylogeny of species that share the same leaf is not necessarily well resolved, but this fact is irrelevant here, because, as we shall see below, the test includes at most one sequence per leaf in each tree reconstructed. Including more than one species per leaf is merely a way to include more data in the test. The eukaryotic reference tree follows the NCBI taxonomy, the bacterial one follows the lineage tree by Bern *et al.* [36] and the fungal reference tree follows the NCBI tree, but with correction regarding the placement of the two *Candida* species [37].

In each trial, a starting sequence from a random species in the innermost leaf is randomly chosen. Then, for each project under scrutiny, we try to build a set of sequences consisting of one ortholog per leaf. If a project predicts more than one sequence orthologous to the starting sequence in a leaf, one of them is picked randomly. If a project predicts no ortholog in a particular leaf, sequence from that leaf are excluded from other projects as well, such that the resulting sets of sequences are of the same size for all projects. If the orthologous groups have less than 5 sequences, the procedure restarts with another starting sequence. Else, based on each orthologous set, we build a tree (as described below) and assess its agreement with the reference species tree by computing the fraction of correct splits derived from the Robinson-Foulds metric [23].

The “comb” structure of the topology is necessary to ensure that a set of sequences orthologous to a starting sequence indeed constitutes an orthologous groups (that is, a set of sequences in which every pair is orthologous): recall that two sequences are orthologs if they split through speciation. Thus, if all bifurcations in the gene trees are speciation events, the set of sequences constitute an orthologous group. Due to the particular topology, each bifurcation is the split of the innermost sequence from another sequence. Since the innermost sequence is orthologous to all other sequences, all bifurcations are speciation events, and the conclusion follows.

### Darwin Least-Squares Distance Trees

The sequences are aligned pairwise using Smith and Waterman [35], with joint ML estimation of all pairwise distances using the *Align* function of Darwin [38]. The estimated distance and variances are used to compute a least-squares distance tree using Darwin's *LeastSquaresTree* function.

### Muscle and RaxML

As a second method for computing the gene tree, we use Muscle [39] as multiple sequence alignment tool in combination with RaxML-VI-HPC version 2.2.3 [40] as tree building package. RaxML builds maximum-likelihood trees. Muscle was run with default parameters, while RaxML was run with *JTT* with 4 gamma categories as amino acid substitution model. The method is repeated from ten random start topologies. The tree with the highest likelihood is taken as the resulting tree of this method.

### Benchmarks from Literature

We used four different sources of manually curated orthology reference sets from the literature: (1) A reconciled tree of Pfam adenosine/AMP deaminase family (PF00962) produced by Engelhardt *et al.* [26],[41]. This tree contains 251 proteins from which we could map 146. (2) Results from detailed phylogenetic analysis on three different COGs presented in [24]. From the originally 116 proteins, 82 were mappable, again restricting on identical sequences. (3) Resulting trees from the phylogenetic analysis by Hughes [27] of 10 gene families. 33 of 165 proteins could be mapped. (4) The ortholog reference set proposed by Hulsen *et al.* [9]. From there 102 of the 167 proteins could be mapped.

For every of those difficult phylogenies, we extracted the orthologous and paralogous relations. For the purpose of this study, those assignments are considered to be error free and are taken as a reference set. For every possible protein pair where both proteins are present in the common set of sequences, we determined whether the project made a true positive, a true negative, a false-positive or a false-negative prediction. Those measurements are then used to infer the true positive and the false-positive rate respectively by taking a Bayesian approach with a uniform prior. Finally, the results of the performance on the four phylogenies have been averaged.

### Functional Based Definition

#### Gene ontology

GO terms and their evidence codes are obtained from EBI and Ensembl for all available species. 255 806 proteins had at least one annotation. Since most annotations are automatically obtained from sequence similarity and all the orthology projects base their predictions on sequence similarity, we only keep the annotations inferred experimentally (Evidence codes *EXP,IDA,IEP,IGI,IMP,IPI*). We end up with 26 676 proteins having 78 912 annotations in total. The similarity between two annotated proteins 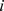 and 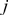 having GO terms 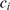 and 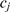 is computed as proposed by Lin [29]

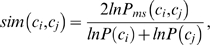
where 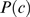 is the probability of encountering the term 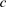 and
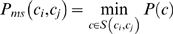
is the probability of the minimum subsummer (or most specific parent) between term 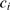 and 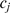. The similarity score obviously varies between 0 (unrelated) and 1 (identical terms). The occurrence probability of GO term 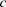 is estimated from the occurrence frequency of GO term 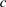 or a child term of 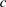 for any instance of a protein intersection set independently.

Proteins are often annotated with multiple GO terms. In such situations, the similarities need to be combined. We follow the rationale of Lord *et al.* [42] and average all the possible similarity values between putative orthologs 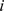 and 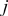, since in general a protein has all the attributed roles. Thus the overall similarity between proteins 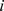 and 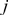 each having its set of GO terms 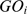 and 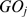 is
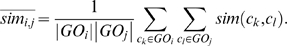



The mean similarity of a project given a (intersection) set of proteins that we show in Figure 4A is the mean similarity between all the putative orthologs stated by the project in the given set of proteins.

#### Enzyme classification

The Swiss Institute of Bioinformatics operates a database on Enzyme nomenclature [30]. In this study we use the release from Nov. 13 2007 of the database. As a first step, we remove all the proteins that are assigned to more than one EC number (3.83%). Then, the proteins from the EC database are mapped to OMA (61518 proteins or 71.16%). For those proteins, we computed the ratio of putative orthologs that map to the same EC class.

#### Correlation in expression profiles

MAS 5.0 processed tissue expression data from human and mouse Affymetric microarray chips (human:U133A/GNF1H; mouse:GNF1M) and the gene mappings as used by Liao and Zhang [31] have been provided by the authors. A total of 25854 probe sets could be mapped to 16295 proteins in the human genome and 17872 probe sets to 15522 mouse proteins. As a measure for the accuracy of the orthology predictions, we computed the average Pearson correlation coefficient of the relative abundance level 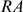 between the putative human and mouse orthologs with respect to the projects' common sequences sets. The relative abundance level of gene 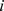 and tissue 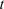 is defined as the relative expression signal intensity in tissue 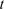, thus
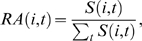
and the correlation between two putative orthologs 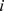 and 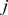 having 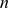 tissues in common
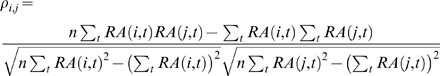



#### Gene neighborhood conservation

The conservation of gene order is measured in the following way. We use the coding sequence features (CDS) from OMA's genome sources (mainly Ensembl, Genome Reviews and EMBL) to determine the order of the genes in the genome. Overlapping genes are excluded, as the order is not resolved. For every predicted orthologous protein pair, we check whether their directly adjacent neighbors (if present) are orthologous too. The verification is performed using the union of all predictions. This ensures that projects with many ortholog predictions are not advantaged over more stringent ones. Whenever we find at least one of the four possible neighbor configurations in the union, we conclude that the neighborhood is conserved.

Formally, the average conservation is
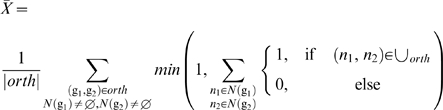
where 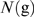 are the neighbors of gene 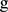 in the projects' common set of proteins, 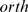 is the set of orthologous pairs and 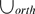 the union of the ortholog predictions.

## Supporting Information

Dataset S1 Fasta formated protein sequences used in the intersection set of the phylogenetic test with Fungi. Part 1 of 4.
(10.22 MB GZ)

Dataset S2 Fasta formated protein sequences used in the intersection set of the phylogenetic test with Fungi. Part 2 of 4.
(10.20 MB GZ)

Dataset S3 Fasta formated protein sequences used in the intersection set of the phylogenetic test with Fungi. Part 3 of 4.
(10.25 MB GZ)

Dataset S4 Fasta formated protein sequences used in the intersection set of the phylogenetic test with Fungi. Part 4 of 4.
(9.20 MB GZ)

Dataset S5 Fasta formated protein sequences used in the intersection set of the phylogenetic test with Eukaryota. Part 1 of 3.
(10.21 MB GZ)

Dataset S6 Fasta formated protein sequences used in the intersection set of the phylogenetic test with Eukaryota. Part 2 of 3.
(10.12 MB GZ)

Dataset S7 Fasta formated protein sequences used in the intersection set of the phylogenetic test with Eukaryota. Part 3 of 3.
(3.69 MB GZ)

Dataset S8 Fasta formated protein sequences used in the intersection set of the phylogenetic test with Bacteria. Part 1 of 3.
(10.18 MB GZ)

Dataset S9 Fasta formated protein sequences used in the intersection set of the phylogenetic test with Bacteria. Part 2 of 3.
(10.12 MB GZ)

Dataset S10 Fasta formated protein sequences used in the intersection set of the phylogenetic test with Bacteria. Part 3 of 3.
(4.45 MB GZ)

Dataset S11 Fasta formated protein sequences used in the intersection set of all the functional based tests. Part 1 of 2.
(6.05 MB GZ)

Dataset S12 Fasta formated protein sequences used in the intersection set of all the functional based tests. Part 2 of 2.
(5.39 MB GZ)

Figure S1 In- and out-paralogy: for instance genes b_1_ and c_2_ are in-paralogs with respect to speciation S_1_, but are out-paralogs with respect to speciation S_2_. Group of orthologs: In such a case, it is not possible to partition the genes into groups of orthologs and in-paralogs with respect to the last speciation event (S_2_). Indeed, a is orthologous to all other genes, but they do not form a group because every other pair is out-paralogous with respect to speciation S_2_.
(0.70 MB TIF)

Figure S2 Results of phylogenetic test using least-squares distance tree: The mean fraction of correct splits (bipartitions) of least-squares distance trees of putative orthologs within three different kingdoms are shown. The higher the value, the better the gene trees agree with the species tree. On the left, the pairwise results between every project and OMA are shown, whereas on the right, the result for the comparison on the common set of proteins of a larger number of projects is shown. Note that the pairwise project comparisons are made based on varying protein sets, and thus cannot be compared to each other. Error bars indicate the 95% confidence intervals of the estimated means. Projects with too little appropriate data could not be evaluated, which explains absent bars. Although not relevant to the present analysis, the fact that a distance-based method reconstructed on average more accurately eukaryotic trees than an ML method goes against the common belief that ML tree building is the more accurate tree reconstruction method. This could be the subject of further investigation.
(2.47 MB TIF)

Table S1 Overview of some project mapping key numbers. Indicated are the number of species, the number of proteins, the average number of orthologs per protein and the number of orthologs per protein normalized by the number of species for the original and the mapped data. We see that the mapped data constitute a reasonable sample of the original data.
(0.02 MB PDF)

Table S2 Overview of the ortholog predictions. In the first column, the number of ortholog predictions made only by the project, in the second the number of common predictions made by the project and OMA and in the third column, the number of predictions made only by OMA are shown.
(0.01 MB PDF)

Text S1 Reference Tree Topologies and Species List: Background data for phylogenetic test
(0.03 MB PDF)

Text S2 Raw Tests Results: Tables of all results with absolute numbers and confidence intervals.
(0.02 MB TXT)

## References

[1] Tatusov RL, Koonin EV, Lipman DJ (1997). A genomic perspective on protein families.. Science.

[2] Remm M, Storm C, Sonnhammer E (2001). Automatic clustering of orthologs and in-paralogs from pairwise
species comparisons.. J Mol Biol.

[3] Li L, Stoeckert CJJ, Roos DS (2003). Orthomcl: identification of ortholog groups for eukaryotic
genomes.. Genome Res.

[4] Dessimoz C, Cannarozzi G, Gil M, Margadant D, Roth A, McLysath A, Huson DH (2005). OMA, a comprehensive, automated project for the identification of
orthologs from complete genome data: Introduction and first achievements.. RECOMB 2005 Workshop on Comparative Genomics.

[5] DeLuca TF, Wu IH, Pu J, Monaghan T, Peshkin L (2006). Roundup: a multi-genome repository of orthologs and evolutionary
distances.. Bioinformatics.

[6] Wheeler DL, Barrett T, Benson DA, Bryant SH, Canese K (2007). Database resources of the National Center for Biotechnology
Information.. Nucleic Acids Res.

[7] Hubbard TJP, Aken BL, Beal K, Ballester B, Caccamo M (2007). Ensembl 2007.. Nucleic Acids Res.

[8] Jensen LJ, Julien P, Kuhn M, von Mering C, Muller J (2008). eggNOG: automated construction and annotation of orthologous
groups of genes.. Nucleic Acids Res.

[9] Hulsen T, Huynen MA, de Vlieg J, Groenen PM (2006). Benchmarking ortholog identification methods using functional
genomics data.. Genome Biol.

[10] Chen F, Mackey AJ, Vermunt JK, Roos DS (2007). Assessing performance of orthology detection strategies applied
to eukaryotic genomes.. PLoS ONE.

[11] Ouzounis C (1999). Orthology: another terminology muddle.. Trends Genet.

[12] Fitch W (2000). Homology a personal view on some of the problems.. Trends Genet.

[13] Jensen RA (2001). Orthologs and paralogs – we need to get it right.. Genome Biol.

[14] Fitch WM (1970). Distinguishing homologous from analogous proteins.. Syst Zool.

[15] Marron M, Swenson KM, Moret BME (2004). Genomic distances under deletions and insertions.. Theor Comput Sci.

[16] Azevedo C, Sadanandom A, Kitagawa K, Freialdenhoven A, Shirasu K (2002). The RAR1 interactor SGT1, an essential component of R
gene-triggered disease resistance.. Science.

[17] Elliott C, Zhou F, Spielmeyer W, Panstruga R, Schulze-Lefert P (2002). Functional conservation of wheat and rice Mlo orthologs in
defense modulation to the powdery mildew fungus.. Mol Plant Microbe Interact.

[18] Tatusov RL, Fedorova ND, Jackson JD, Jacobs AR, Kiryutin B (2003). The cog database: an updated version includes eukaryotes.. BMC Bioinformatics.

[19] Berglund A, Sjölund E, Ostlund G, Sonnhammer E (2008). InParanoid 6: eukaryotic ortholog clusters with inparalogs.. Nucleic Acids Res.

[20] Roth AC, Dessimoz C, Gonnet GH (2008). The algorithm of OMA, large-scale orthology inference.. BMC Bioinformatics.

[21] Overbeek R, Fonstein M, D'Souza M, Pusch GD, Maltsev N (1999). The use of gene clusters to infer functional coupling.. Proc Natl Acad Sci U S A.

[22] Wall DP, Fraser HB, Hirsh AE (2003). Detecting putative orthologs.. Bioinformatics.

[23] Robinson DF, Foulds LR (1981). Comparison of phylogenetic trees.. Math Biosci.

[24] Dessimoz C, Boeckmann B, Roth A, Gonnet GH (2006). Detecting non-orthology in the cog database and other approaches
grouping orthologs using genome-specific best hits.. Nucleic Acids Res.

[25] van der Heijden RT, Snel B, van Noort V, Huynen MA (2007). Orthology prediction at scalable resolution by phylogenetic tree
analysis.. BMC Bioinformatics.

[26] Engelhardt BE, Jordan MI, Brenner SE, Cohen WW, Moore A (2006). A graphical model for predicting protein molecular function.. ICML 2006: Proceedings of the 23th International Conference on Machine
Learning.

[27] Hughes AL (1998). Phylogenetic tests of the hypothesis of block duplication of
homologous genes on human chromosomes 6, 9, and 1.. Mol Biol Evol.

[28] Harris MA, Clark J, Ireland A, Lomax J, Ashburner M (2004). The Gene Ontology (GO) database and informatics resource.. Nucleic Acids Res.

[29] Lin D (1998). An information-theoretic definition of similarity.. Proc. 15th International Conf. on Machine Learning.

[30] Bairoch A (2000). The enzyme database in 2000.. Nucleic Acids Res.

[31] Liao BY, Zhang J (2006). Evolutionary conservation of expression profiles between human
and mouse orthologous genes.. Mol Biol Evol.

[32] Notebaart RA, Huynen MA, Teusink B, Siezen RJ, Snel B (2005). Correlation between sequence conservation and the genomic context
after gene duplication.. Nucleic Acids Res.

[33] Bandyopadhyay S, Sharan R, Ideker T (2006). Systematic identification of functional orthologs based on
protein network comparison.. Genome Res.

[34] Dost B, Shlomi T, Gupta N, Ruppin E, Bafna V, Speed TP, Huang H (2007). Qnet: A tool for querying protein interaction networks.. RECOMB.

[35] Smith TF, Waterman MS (1981). Identification of common molecular subsequences.. J Mol Biol.

[36] Bern M, Goldberg D, Lyashenko E (2006). Data mining for proteins characteristic of clades.. Nucleic Acids Res.

[37] Wong S, Fares M, Zimmermann W, Butler G, Wolfe K (2003). Evidence from comparative genomics for a complete sexual cycle in
the ‘asexual’ pathogenic yeast candida glabrata.. Genome Biol.

[38] Gonnet GH, Hallett MT, Korostensky C, Bernardin L (2000). Darwin v. 2.0: An interpreted computer language for the
biosciences.. Bioinformatics.

[39] Edgar RC (2004). Muscle: a multiple sequence alignment method with reduced time
and space complexity.. BMC Bioinformatics.

[40] Stamatakis A (2006). RAxML-VI-HPC: maximum likelihood-based phylogenetic analyses with
thousands of taxa and mixed models.. Bioinformatics.

[41] Engelhardt BE, Jordan MI, Muratore KE, Brenner SE (2005). Protein molecular function prediction by Bayesian phylogenomics.. PLoS Comput Biol.

[42] Lord PW, Stevens RD, Brass A, Goble CA (2003). Investigating semantic similarity measures across the gene
ontology: the relationship between sequence and annotation.. Bioinformatics.

