# Supplementary figures and images for: Phylogenetic and Functional Assessment of Orthologs Inference Projects and Methods
Source: PLoS Comput Biol. 2009 Jan 16;5(1):e1000262. doi: 10.1371/journal.pcbi.1000262 (PMC2612752; doi:10.1371/journal.pcbi.1000262)

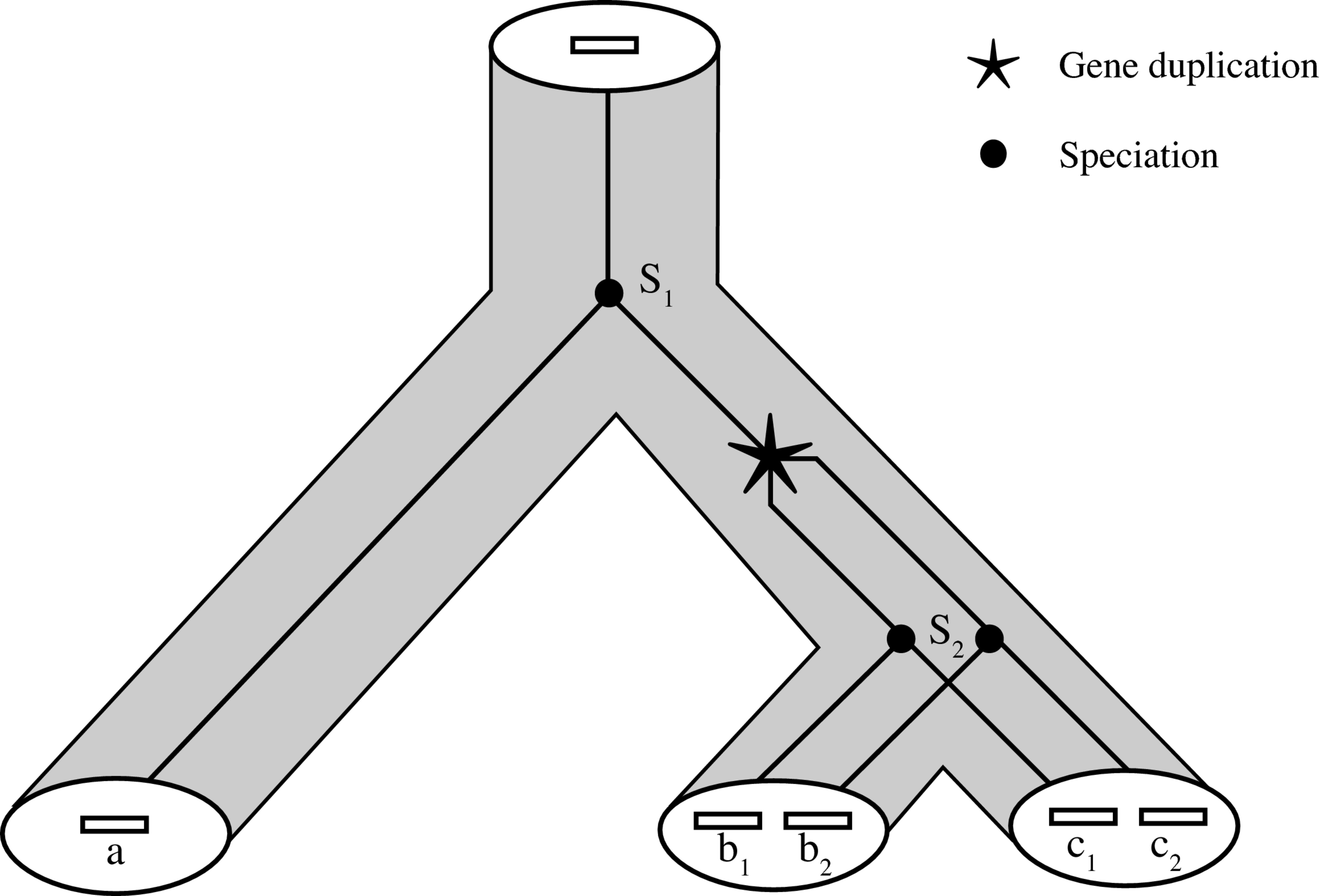

Supplement: Figure S1 — In- and out-paralogy: for instance genes b1 and c2 are in-paralogs with respect to speciation S1, but are out-paralogs with respect to speciation S2. Group of orthologs: In such a case, it is not possible to partition the genes into groups of orthologs and in-paralogs with respect to the last speciation event (S2). Indeed, a is orthologous to all other genes, but they do not form a group because every other pair is out-paralogous with respect to speciation S2. (0.70 MB TIF) [file pcbi.1000262.s013.tif]

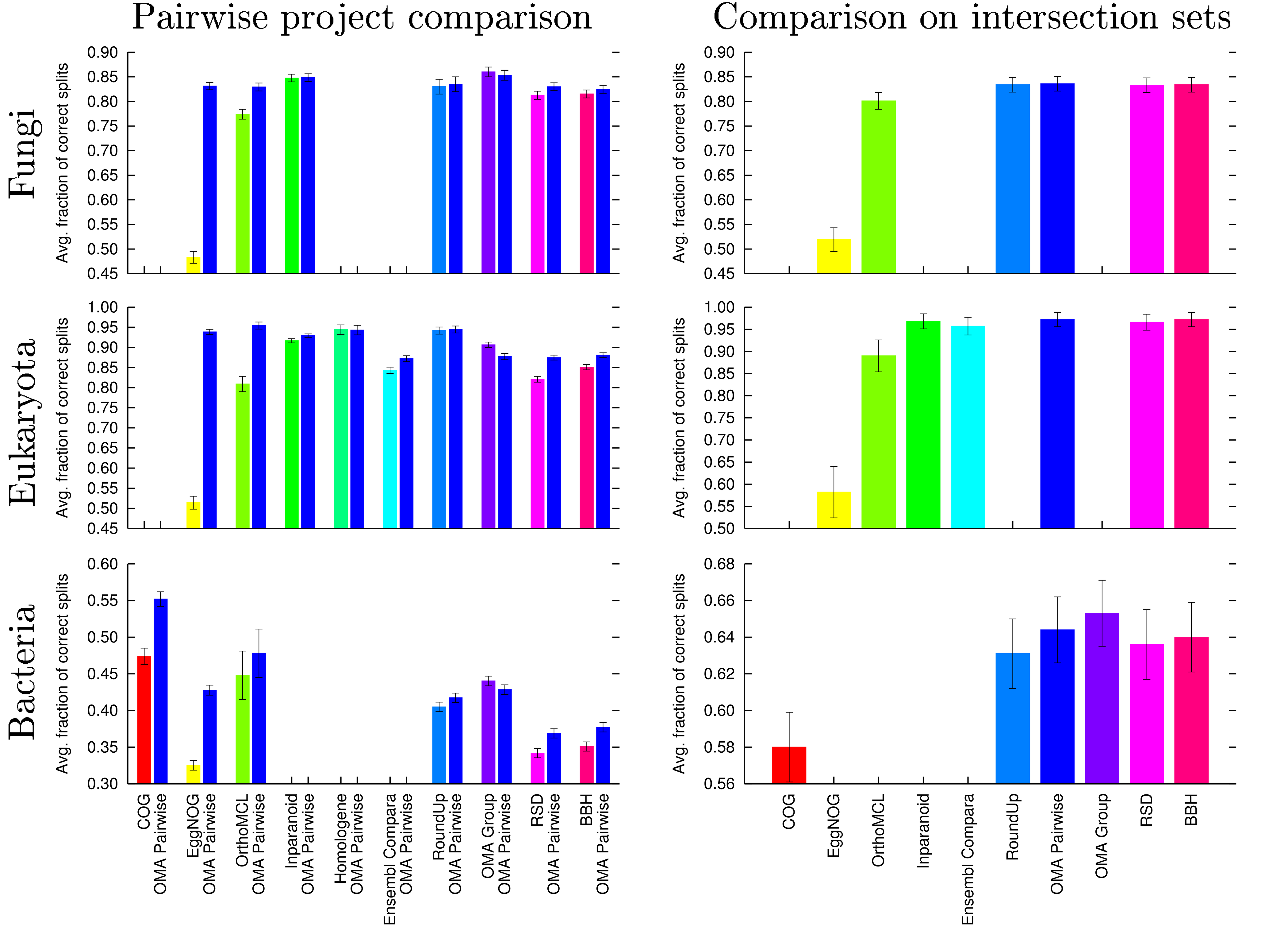

Supplement: Figure S2 — Results of phylogenetic test using least-squares distance tree: The mean fraction of correct splits (bipartitions) of least-squares distance trees of putative orthologs within three different kingdoms are shown. The higher the value, the better the gene trees agree with the species tree. On the left, the pairwise results between every project and OMA are shown, whereas on the right, the result for the comparison on the common set of proteins of a larger number of projects is shown. Note that the pairwise project comparisons are made based on varying protein sets, and thus cannot be compared to each other. Error bars indicate the 95% confidence intervals of the estimated means. Projects with too little appropriate data could not be evaluated, which explains absent bars. Although not relevant to the present analysis, the fact that a distance-based method reconstructed on average more accurately eukaryotic trees than an ML method goes against the common belief that ML tree building is the more accurate tree reconstruction method. This could be the subject of further investigation. (2.47 MB TIF) [file pcbi.1000262.s014.tif]
