# Supplementary material for: Phylogenetic and Functional Assessment of Orthologs Inference Projects and Methods
Source: PLoS Comput Biol. 2009 Jan 16;5(1):e1000262. doi: 10.1371/journal.pcbi.1000262 (PMC2612752; doi:10.1371/journal.pcbi.1000262)
Supplement: Table S1 — Overview of some project mapping key numbers. Indicated are the number of species, the number of proteins, the average number of orthologs per protein and the number of orthologs per protein normalized by the number of species for the original and the mapped data. We see that the mapped data constitute a reasonable sample of the original data. (0.02 MB PDF) [file pcbi.1000262.s015.pdf]

Projects	Original data				Mapped data				
	species	sequences	$\frac{pred}{seq}$	$\frac{pred}{seq(n-1)}$	species	sequences	pred	$\frac{pred}{seq}$	$\frac{pred}{seq(n-1)}$
COG	66	192987	90.3	1.389	63	81.5%	66.2%	73.4	1.184
KOG	7	112920	21.2	3.541	7	50.0%	24.2%	10.3	1.713
EggNOG	373	1513782	702.9	1.889	190	47.1%	21.1%	314.4	1.664
OrthoMCL	56	632455	16.1	0.293	46	45.0%	25.1%	9.0	0.199
Inparanoid	35	610047	29.5	0.868	33	69.6%	42.6%	18.1	0.565
Homologene	19	198758	6.2	0.346	17	51.1%	28.4%	3.5	0.216
Ensembl Compara	35	792504	19.2	0.566	35	74.0%	80.1%	20.8	0.612
RoundUp	220	1152167	21.6	0.099	210	67.3%	78.7%	25.3	0.121
OMA Pairwise	550	2477966	62.6	0.114					
OMA Group	550	2477966	28.9	0.053					
RSD	550	2477966	78.4	0.143					
BBH	550	2477966	77.7	0.141					

Table S1: Overview of some project mapping key numbers. Indicated are the number of species, the number of proteins, the average number of orthologs per protein and the number of orthologs per protein normalized by the number of species for the original and the mapped data. We see that the mapped data constitute a reasonable sample of the original data.
