# Supplementary material for: Phylogenetic and Functional Assessment of Orthologs Inference Projects and Methods
Source: PLoS Comput Biol. 2009 Jan 16;5(1):e1000262. doi: 10.1371/journal.pcbi.1000262 (PMC2612752; doi:10.1371/journal.pcbi.1000262)
Supplement: Table S2 — Overview of the ortholog predictions. In the first column, the number of ortholog predictions made only by the project, in the second the number of common predictions made by the project and OMA and in the third column, the number of predictions made only by OMA are shown. (0.01 MB PDF) [file pcbi.1000262.s016.pdf]

Projects	$\|P \backslash O\|$	$\|P \cap O\|$	$\|O \backslash P\|$
COG	5 119 005	649 108	13 293
KOG	265 206	24 922	1 010
EggNOG	103 865 058	8 258 006	153 258
OrthoMCL	745 034	532 408	84 133
Inparanoid	2 311 305	1 529 041	247 619
Homologene	40 901	134 976	74 412
Ensembl Compara	2 980 740	3 122 685	956 591
RoundUp	2 628 480	7 171 594	2 628 028
OMA Group	0	35 817 244	41 691 785
RSD	36 213 554	60 901 575	16 607 454
BBH	32 883 589	63 330 979	14 178 050

Table S2: Overview of the ortholog predictions In the first column, the number of ortholog predictions made only by the project, in the second the number of common predictions made by the project and OMA and in the third column, the number of predictions made only by OMA are shown.
