# Supplementary material for: Phylogenetic and Functional Assessment of Orthologs Inference Projects and Methods
Source: PLoS Comput Biol. 2009 Jan 16;5(1):e1000262. doi: 10.1371/journal.pcbi.1000262 (PMC2612752; doi:10.1371/journal.pcbi.1000262)
Supplement: Text S1 — Reference Tree Topologies and Species List: Background data for phylogenetic test (0.03 MB PDF) [file pcbi.1000262.s017.pdf]

Supplementary material for phylogenetic based test

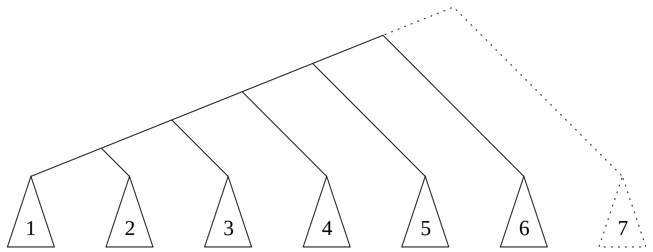

The figure shows the topology of the used reference trees. The numbers in the leaves are placeholders for the species used. They coincide with the numbers of following species listing. For species identification, we use the five letter abbreviations which is available from http://www.expasy.ch/cgi-bin/speclist.

Eukaryota tree

1. *Homo sapiens:* HUMAN
2. *Other Primates:* MACMU, OTOGA, PANTR
3. *Other Mammalia:* BOVIN, CANFA, CAVPO, DASNO, ECHTE, ERIEU, FELCA, LOXAF, MONDO, MOUSE, MYOLU, ORNAN, RABIT, RATNO, SORAR, SPETR, TUPGB
4. *Other Vertebrata:* CHICK, DANRE, FUGRU, GASAC, ORYLA, TETNG, XENTR
5. *Protostomia:* AEDAE, ANOGA, APIME, DROME, DROPS
6. *Fungi:* ASHGO, ASPFU, BOTCI, CANAL, CANGA, CRYNE, DEBHA, ENCCU, KLULA, LODEL, MAGGR, PHANO, PICST, SCHPO, USTMA, YARLI, YEAST

Fungi tree

1. YEAST, CANGA
2. ASHGO, KLULA
3. CANAL, DEBHA
4. YARLI
5. ASPFU, SCHPO
6. CRYNE, ENCCU

Bacteria tree

1. *Gammaproteobacteria:* ACIAD, ACIBT, ACTP2, AERHH, AERS4, ALCBS, ALHEH, BAUCH, BLOFL, BLOPB, BUCAI, BUCAP, BUCBP, BUCCC, CARRP, CHRSD, COLP3, COXBU, DICNV, ECO57, ECOK1, ECOL5, ECOL6, ECOLI, ECOUT, ERWCT, FRAT1, FRATH, FRATN, FRATO, FRATT, FRATW, HAEDU, HAEI8, HAEIE, HAEIG, HAEIN, HAES1, HAHCH, HALHL, IDILO, LEGPA, LEGPC, LEGPH, LEGPL, MANSM, MARAV, METCA, NITOC, PASMU, PHOLL, PHOPR, PSE14, PSEA6, PSEAB, PSEAE, PSEE4, PSEF5, PSEHT, PSEPF, PSEPK, PSESM, PSEU2, PSEU5, PSYAR, PSYCK, PSYIN, RUTMC, SACD2, SALCH, SALPA, SALTI, SALTY, SHEAM, SHEDO, SHEFN, SHELP, SHEON, SHESA, SHESM, SHESR, SHESW, SHIBS, SHIDS, SHIF8, SHIFL, SHISS, SODGM, THICR, VESOH, VIBCH, VIBF1, VIBPA, VIBVU, VIBVY, WIGBR, XANAC, XANC5, XANC8, XANCP, XANOM, XANOR, XYLFA, XYLFT, YERE8, YERPA, YERPE, YERPN, YERPP, YERPS

2. *Betaproteobacteria:* ACIAC, ACISJ, AZOSB, AZOSE, BORA1, BORBR, BORPA, BORPE, BURCA, BURCH, BURCM, BURMA, BURP0, BURP1, BURPS, BURS3, BURTA, BURXL, CHRVO, DECAR, HERAR, JANMA, METFK, METPP, NEIG1, NEIMA, NEIMB, NEIMF, NITEC, NITEU, NITMU, POLNA, POLSJ, RALEH, RALEJ, RALME, RALSO, RHOFD, THIDA, VEREI

3. *Alphaproteobacteria:* AGRT5, ANAMM, ANAPZ, BARBK, BARHE, BARQU, BRAJA, BRASB, BRASO, BRUA2, BRUAB, BRUME, BRUO2, BRUSU, CAUCR, EHRCJ, EHRCR, EHRRG, EHRRW, ERYLH, GLUOX, GRABC, HYPNA, JANSC, MAGMM, MARMM, MESSB, NEOSM, NITHX, NITWN, NOVAD, ORITB, PARDP, PELUB, RHIEC, RHIL3, RHILO, RHIME, RHOP2, RHOP5, RHOPA, RHOPB, RHOPS, RHORT, RHOS1, RHOS4, RICBR, RICCN, RICFE, RICPR, RICTY, ROSDO, SILPO, SILST, SPHAL, WOLPM, WOLTR, ZYMMO

4. *Deltaproteobacteria:* ANADE, BDEBA, DESDG, DESPS, DESVH, DESVV, GEOMG, GEOSL, LAWIP, MYXXD, PELCD, PELPD, SYNAS, SYNFM

5. *Epsilonproteobacteria:* CAMFF, CAMJE, CAMJJ, CAMJR, HELAH, HELHP, HELPH, HELPJ, HELPY, NITSB, SULNB, THIDN, WOLSU

6. *Spirochaetes:* BORAP, BORBU, BORGA, LEPBJ, LEPBL, LEPIC, LEPIN, TREDE, TREPA

7. *Firmicutes:* AYWBP, BACAH, BACAN, BACC1, BACCR, BACCZ, BACHD, BACHK, BACLD, BACSK, BACSU, CARHZ, CLOAB, CLOD6, CLONN, CLOP1, CLOPE, CLOPS, CLOTE, CLOTH, DESHY, ENTFA, GEOKA, GEOTN, LACAC, LACBA, LACC3, LACDA, LACDB, LACGA, LACJO, LACLA, LACLM, LACLS, LACPL, LACS1, LACSS, LEUMM, LISIN,

LISMF, LISMO, LISW6, MESFL, MOOTA, MYCCT, MYCGA, MYCGE, MYCH2, MYCH7, MYCHJ, MYCMO, MYCMS, MYCPE, MYCPN, MYCPU, MYCS5, OCEIH, OENOB, ONYPE, PEDPA, STAA3, STAA8, STAAB, STAAC, STAAM, STAAN, STAAR, STAAS, STAAW, STAEQ, STAES, STAHJ, STAS1, STRA1, STRA3, STRA5, STRMU, STRP1, STRP2, STRP3, STRP6, STRP8, STRPB, STRPC, STRPD, STRPF, STRPG, STRPM, STRPN, STRR6, STRS2, STRSV, STRSY, STRT1, STRT2, STRTD, SYNWW, THETN, UREPA
